# Do Culture-Negative Periprosthetic Joint Infections Have a Worse Outcome Than Culture-Positive Periprosthetic Joint Infections? A Systematic Review and Meta-Analysis

**DOI:** 10.1155/2018/6278012

**Published:** 2018-07-12

**Authors:** Marie Reisener, Carsten Perka

**Affiliations:** Department of Orthopedics, Charité University Hospital, Berlin, Germany

## Abstract

**Background:**

Culture-negative periprosthetic joint infections (CN PJI) have not been well studied, and due to the lack of consensus on PJI, especially with culture-negative infections, there are considerable uncertainties. Due to the challenging clinical issue of CN PJI the aim of this systematic review is to describe incidence, diagnosis, and treatment outcomes based on the current literature on CN PJI.

**Hypothesis:**

The review is designed to assess the formal hypothesis that CN PJI of the hip and knee have a poorer outcome when compared with culture-positive ones.

**Study Design:**

It is systematic review with level of evidence 3.

**Methods:**

EMBASE, MEDLINE, and the Cochrane Library were searched electronically in January 2018. All studies regarding CN PJI of the hip or knee published in English or German with a minimum of 10 patients were included. Afterwards, the authors performed a descriptive analysis of diagnosis and treatment outcome.

**Result:**

Eight studies were identified that met the inclusion criteria. The incidence of CN PJI in the hip or knee ranged from 7% to 42 %. The included studies were pooled to give an overall incidence rate estimate of 11 % [95% confidence interval (CI): 10-12] based on a random-effects model. The most common surgical intervention was the two-stage revision of prosthesis with 283 patients. Postoperatively, the majority of patients received vancomycin as the antibiotic treatment, alone or in combination with other antibiotics. The rate of succesfully treated infections varied from 85% to 95 % in all included studies. The two-stage exchange arthroplasty had the best outcome, based on the infection-free survival rate of 95%, five years after treatment.

**Conclusions:**

We conclude that CN PJI have the same or even better results than culture-positive infections. Nonetheless, a standardized diagnostic protocol and evidence-based treatment strategies for CN PJI should be implemented for further studies.

## 1. Introduction

When performing arthroplasty of the hip or knee, periprosthetic joint infections are among the most serious complications after the procedure. 1% of all hip replacements and 2-3% of primary knee prostheses are affected [1, 2]. In the future, a rise in infections is likely due to an increase of implantations, increasing lifespans of patients and the resultant longer prostheses retention times.

Due to a lack of consensus on diagnosis and treatment of periprosthetic joint infections, especially culture-negative infections, there still seem to be considerable uncertainites. Different diagnostic protocols for detecting periprosthetic joint infections have been published, and hence there seems to be no standardized protocol being used across studies [3–13].

Moreover, comparisons of treatment outcomes are difficult to make, as the current evidence does not conclusively support a superior treatment strategy for periprosthetic joint infections.

The culture-negative periprosthetic joint infection is even more demanding in diagnosis and treatment, as without positive culture the uncertainty about the correct diagnosis of infection grows. Without knowing the causing microorganism, it is a challenge to determine the right treatment and choice of antibiotics for any patient. This is all the more difficult due to the sparse existing literature on the treatment and outcome of CN PJI.

This systematic review therefore aims to give an overview on the current database of studies concerning culture-negative periprosthetic joint infections of the hip and knee. The different diagnosis protocols and results after treatment were analyzed, and whether culture-negative infections really have a worse outcome when compared to culture-positive ones was evaluated.

## 2. Material and Methods

In January 2018 the authors conducted a systematic literature search of MEDLINE, EMBASE via OvidSP, and the Cochrane Library addressing culture-negative periprosthetic joint infections. To identify additional studies that possibly fit the criteria and had not been discovered via the electronic database search, the authors reviewed the bibliographies of the chosen studies and review articles. The systematic review has been reported in accordance with the PRISMA statement [14]. See Table 1 for search terms used.

Inclusion criteria comprised studies published in English or German, numbers of patients >10, and studies regarding culture-negative periprosthetic joint infections after arthroplasty of the knee or hip. Although two-stage exchange arthroplasty is the most widely performed procedure, all treatment strategies were included in the search. Studies with prosthetic joint infections of another region than knee or hip were excluded, as well as case reports, review articles, opinion of experts, and letters to the editors. The abstracts of the selected studies were screened. If they were found to be inadequate, the full text was evaluated to determine whether a study was eligible for inclusion. Two of the authors independently carried out the process described above. Lack of consensus was resolved by thorough discussion. A level of evidence based on The Journal of Bone and Joint Surgery guidelines was then assigned to every article. Different variables for a comparative analysis of the outcome of each study were included in a data sheet (Table 2). A descriptive review of the variables, such as the infection control rate and outcome of the included studies, was drafted, and a comparison between all studies was performed. The included studies were pooled to give an overall incidence rate based on a random-effects model with 95% confidence interval (CI). Heterogeneity between the studies was assessed with a chi-square-test and quantified with I^2^ statistics. Publication bias was evaluated with funnel plot analysis.

## 3. Results

**Figure 1:**
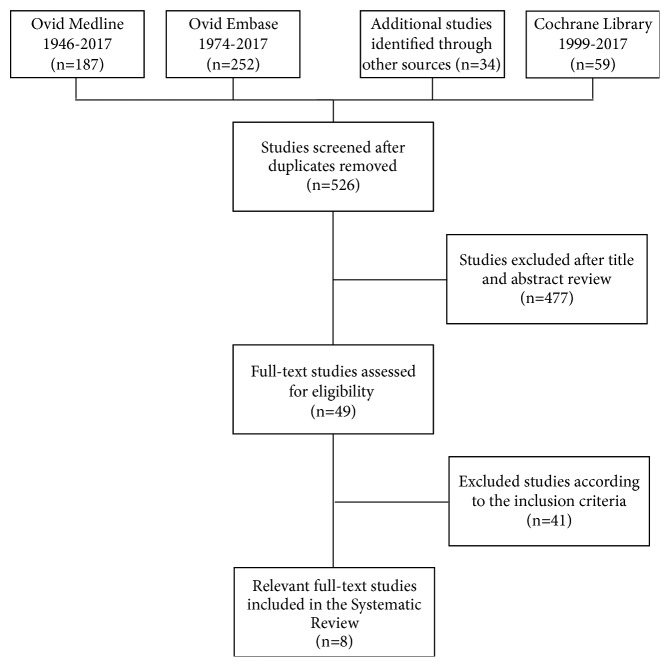
Flow chart.

A flow chart of our literature research was created using the PRISMA (Preferred Reporting Items for Systematic Reviews and Meta-Analyses) guidelines (Figure 1).

532 potential studies matching our inclusion criteria were identified via the search strategy and manual screening of the bibliographies of relevant studies. We excluded 477 studies after reviewing title and abstract. This left 49 full-text studies to be assessed for eligibility. Finally, 8 papers were selected for inclusion in our systematic review and meta-analysis [15–22].

**Figure 2:**
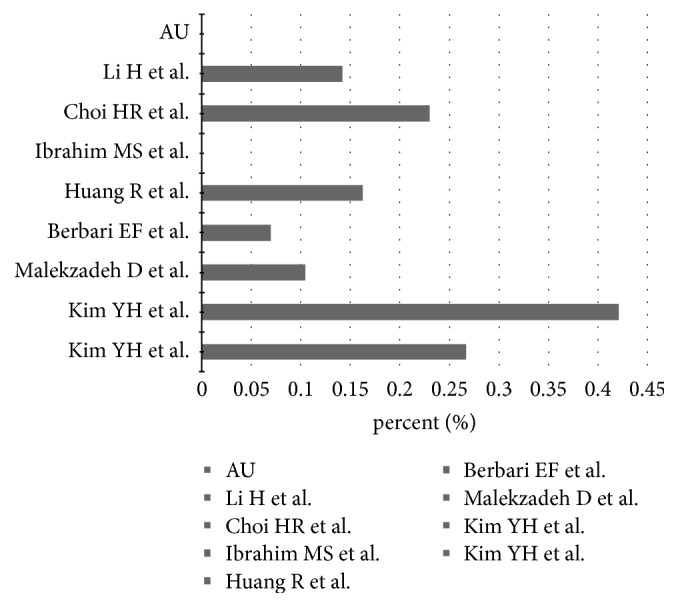
Range of incidence of CN PJI.

**Figure 3:**
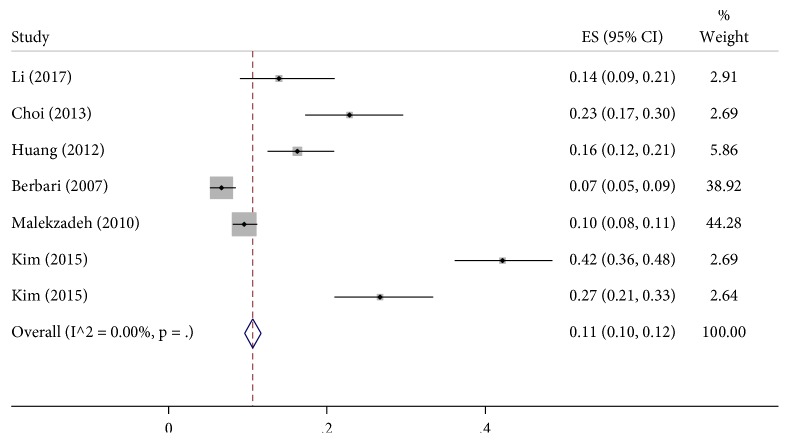
Rates of incidence for culture-negative periprosthetic joint infections of the hip and the knee. Summary estimates for the incidence of CN PJI were calculated using random-effects models with 95% confidence interval (CI). An I^2^ value (statistical heterogeneity) of 0.00% indicates a low variability in intrastudy differences in the overall effect size.

Table 2 shows short summaries of the results of all included studies. All studies have retrospective character and lower quality, with level III of evidence based on The Journal of Bone and Joint Surgery guidelines. All studies were published between 2007 and 2017. The incidence rate of culture-negative periprosthetic infections in the hip or knee ranged from 7% to 42 % with a total number of all included patients being 3,342. Of these, 504 were culture-negative (Figure 2). The included studies were pooled to give an overall incidence rate estimate of 11 % [95% confidence interval (CI): 10-12] based on a random-effects model (Figure 3).

**Figure 4:**
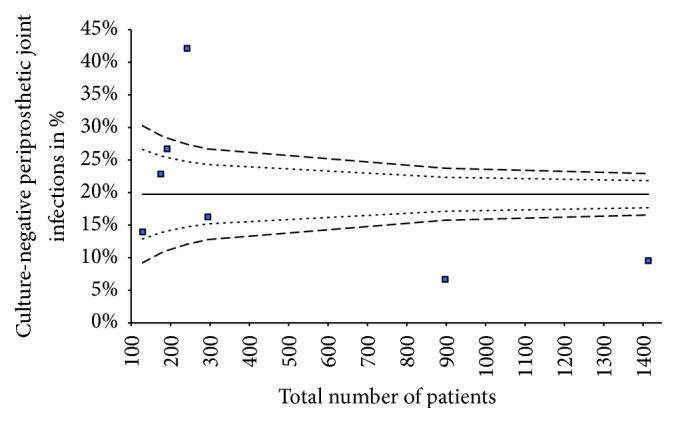
Funnel plot analyses.

**Figure 5:**
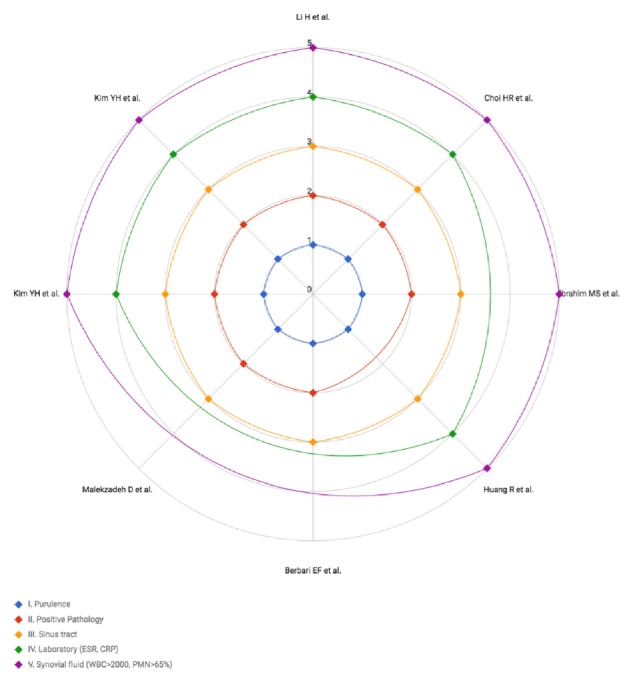
Definition of diagnosis of culture-negative periprosthetic joint infections.

Funnel plot analysis of included studies assessing the overall incidence of CN PJI revealed a publication bias (Figure 4). 36% of all included culture-negative cases were periprosthetic hip infections, and 64% were prosthetic knee infections. A total number of 137 patients were treated for irrigation and debridement with retention of the prosthesis, 16 patients with one-stage exchange arthroplasty, 42 with permanent resection of the joint, and 26 patients with other treatment options like chronic antibiotic suppression. The two-stage revision of prosthesis was the most common surgical intervention with a total number of 283 patients. The studies differ in the diagnostic protocols used to identify culture-negative infections. Often the diagnostic criteria of the Musculoskeletal Infection Society [8] are used as a reference. To better compare the included studies, a graphic was created (Figure 5).

As a postoperative antibiotic, vancomycin was used to treat most of the patients in the included studies, either alone or in combination with other antibiotics. In the studies of Berbari et al. and Malekzadeh et al. cephalosporins were more commonly used to eliminate a periprosthetic joint infection. The relevant studies documented prior use of antibiotics as a risk factor for culture-negative periprosthetic infections.

**Figure 6:**
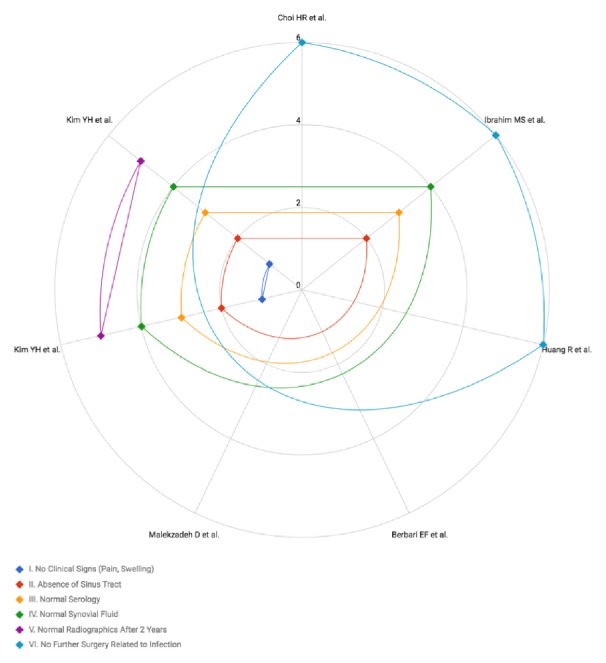
Definition of successful treatment.

The included studies define a successful treatment with variable parameters [15–22]. Intersections of the parameters are illustrated in the following graphic, excluding Li et al. as the study did not specify parameters (Figure 6).

The rate of successful treated infections varied from 85% to 95 % in all included studies. The majority of studies observe infection-free survival rates in 3-year and 5-year time-intervals. The overall infection-free survival rate ranged from 67% to 94%. The two-stage exchange arthroplasty has the best outcome with regard to the infection-free survival rate with rates up to 95% five years after treatment. When comparing the outcomes of culture-negative periprosthetic infections with those of culture-positive periprosthetic infections, all studies came to the conclusion that culture-negative infections have the same or, in the study of Choi et al., even better results than culture-positives.

## 4. Discussion

Periprosthetic joint infections are serious complications that may occur after joint replacement. The incidence ranges from 2% to 3% in primary knee [1, 2] and 1% to 4% in primary hip replacement [2, 24]. In this systematic review, the incidence rate of CN PJI ranged from 7% to 42% [15–22] with a pooled incidence rate of 11%.

The aim of this study is to identify the relevant studies on culture-negative periprosthetic joint infections from the hip and knee and to analyze the reported incidences, diagnostic protocols, and treatment outcomes.

Treating a periprosthetic infection even when the causing organism is known is challenging in itself and a topic of the current investigations [25–29]. When there is no identification of the causing pathogen it is certainly an even bigger challenge. A culture-negative infection is still a subject of controversy because of a lack of literature for a consistent diagnostic protocol and optimal treatment recommendations. Because there are no consistent diagnostic parameters, a comparison between the studies is complicated. While reviewing the literature, the authors found different classifications for the diagnosis of a periprosthetic joint infection (Table 3).

A consistent usage from one classification, separated from the author, joint, or location of the study was not recognizable. Renz and Trampuz et al. published a diagnostic protocol following the international recommendations for usage in further studies to make comparisons between studies and results more reliable (Table 4). In the case that the pathogen cannot be identified, there are three additional parameters to confirm the periprosthetic joint infection.

Reasons for culture-negative periprosthetic joint infections are not definitely resolved. They could include inappropriate diagnostic tools for rare organisms such as mycobacterium, fungi, and others like Brucella or Coxiella burnetti that are difficult to identify using routine methods [15, 16, 30]. The most common risk factor in our systematic review for culture-negative infection was the prior use of antibiotics [15, 18, 22] which can compromise the sensitivity of routinely used diagnostic laboratory tests. For this reason, Della Valle et al. in the clinical practice guideline of American Academy of Orthopedic Surgeons recommends that the antimicrobial treatment be interrupted at least two weeks before aspiration [5]. To increase the detection rate of the low-virulence microorganisms multiple samples (minimum 3) should be taken, and an adequate growth time of at least 14 days [2, 31] should be allowed. Emphasis is placed on new diagnostic tools for improving the sensitivity and specifying for diagnosis of culture-negative prosthetic joint infections, while reducing the number of false-negative results. Trampuz et al. demonstrated the importance of sonication of prostheses in improving diagnosis of periprosthetic joint infections of the knee and the hip, since this method attains more sensitivity than conventional periprosthetic-tissue culture, particularly in patients with prior antibiotic treatment [31]. The most common molecular biological technique is the polymerase chain reaction to detect the causing microorganism [32, 33]. Even unusual species like fungal periprosthetic joint infections could be detected with a selective medium and an increased incubation time [34]. The analyses of the synovial fluid with new biomarkers are currently validated in clinical studies [2]. The alpha-defensin test shows especially good results in detecting a periprosthetic joint infection [2, 35, 36], but it is yet to be validated in larger studies. Next-generation sequencing has recently gained attention and is a topic of current investigations to evaluate the accuracy in identifying causing microorganisms in periprosthetic joint infections, especially in culture-negative infections [37].

The outcome of PJI is determined by the choice of surgical treatment. There are different treatment strategies, including irrigation, debridement, and retention of the prosthesis, one-stage exchange arthroplasty, or two-stage exchange of the prosthesis. The choice of the optimal treatment must be made jointly by orthopedic surgeons and experienced infectologists in accordance with the type of infection and patient's condition.

The largest amount of data in the literature is focused on the two-stage exchange arthroplasty, since this is still considered the gold standard with the lowest reinfection rates, from 0% to 36% [29, 38–44], and best functional outcomes [45–49]. But studies researching the one-stage exchange arthroplasty have also found similar reinfection rates, from 2% to 40% [27, 42, 45, 50–54]. In our systematic review most patients with culture-negative periprosthetic joint infections were treated with two-stage exchange arthroplasty, followed by 4-6 weeks of antibiotic treatment. The two-stage exchange has the highest infection-free survival rate up to 95% after five years of follow-up and a success rate ranging from 70% up to 100%. Of the included studies none recommended one-stage exchange as the first treatment option.

The included studies used different parameters to define a successful treatment. To evaluate and compare the outcome after treatment, a consistent definition of a successful treatment should be determined to enable a reliable comparison between different studies and treatment options.

As was the case regarding the surgical treatment of PJI, there is no consensus in the literature about a standardized protocol for antibiotic usage, especially not in CN PJI. Vancomycin was the antibiotic used to treat most of the patients in our included studies after surgery, either alone or in combination with other antibiotics. Choi et al. reported that high-dosage vancomycin has a better outcome in CN PJI. The rising usage of vancomycin in culture-negative infections may also be encouraged by an increasing number of MRSA infections [13]. Besides the antibiotic agent, the duration of parental and oral antibiotic treatment is another uncertain topic in the published literature, and no treatment protocol has yet been established. Trampuz et al. therefore developed a antimicrobial treatment based on international references [23] (Table 5).

Our systematic review has several limitations. First of all, the included studies are based on level III evidence and retrospective in design, which leads to a limited validity of the results of our study. Secondly, only studies published in English or German were selected, resulting in a selective presentation of included studies and results. Only eight studies that met all inclusion criteria were assessed. This led to a small sample size of patients, resulting in restricted validity of our findings. Furthermore, this only allowed us to perform a descriptive analysis of the data. Due to the small sample size, statistical methods used in the meta-analysis to summarize the results are statistically insignificant. With a low heterogeneity in the incidence rates provided by the studies we included, referral bias possibly affects the results. The possibility of not having retrieved all relevant information published on CN PJI should also be considered as one of the limitations of our study. Further, due to the lack of literature which deals with CN PJI and because of publications focusing only on positive results treating CN PJI, a publication bias is likely. Additionally, the included studies did not utilize a standardized treatment protocol (e.g., different surgeons and operative standards, interval between stages, spacer, antibiotic treatment, and duration), which made a direct comparison of their results difficult. The descriptive analysis could not address the functional status after treatment in the selected studies because of missing information in the primary studies.

When the microorganism is confirmed, treatment outcomes are well documented in the literature. However, treatment outcome of culture-negative PJI is only reported in a few studies. In all eight studies included in this systematic review, the clinical outcome and infection control rates are similar to CP PJI groups or have even higher rates of successful treatments [16]. At the same time, when assessing the treatment success of CN PJI, one should consider the relatively short follow-up of the included studies.

Also one of the recently published articles comparing the outcome of culture-negative to culture-positive periprosthetic joint infections Kang et al. came to the conclusion that CN PJI can be treated successfully and can even show a better outcome regarding clinical course [55].

In conclusion, a culture-negative status may not be a negative prognostic factor for treatment outcome. One clearly significant factor is the appropriate selection of the surgical and antimicrobial treatment according to the type of infection, including additional factors like comorbidities, status of the patient, and operative risk for the patient. To increase the validity of the conclusions in further studies, prospectively designed studies of culture-negative PJI should implement a standardized diagnostic protocol and evidence-based treatment strategies for culture-negative periprosthetic joint infections. This will significantly increase the commensurability and thus yield more tangible recommendations.

## Figures and Tables

**Table 1:** Search strategy.

**Search #**	**Query**
#1	periprosthetic infection or periprosthetic joint infection or surgical wound infection or prosthesis-related infection

#2	knee arthroplasty or total knee arthroplasty or knee replacement or knee prosthesis or arthroplasty, replacement, knee

#3	hip arthroplasty or total hip arthroplasty or hip replacement or hip prosthesis or arthroplasty, replacement, hip

#4	Culture negative OR culture

#6	#1 AND #2 AND #4

#7	#1 AND #3 AND #4

**Table 2:** Data sheet for comparative analysis [15–22].

Author	Li H et al.	Choi HR et al.	Ibrahim MS et al.	Huang R et al.	Berbari EF et al.	Malekzadeh D et al.	Kim YH et al.	Kim YH et al.
Title	Two-stage revisions for culture-negative infected total knee arthroplasties: A five-year outcome in comparison with one-stage and two-stage revisions for culture-positive cases.	Periprosthetic joint infection with negative culture results: Clinical characteristics and treatment outcome.	Two-stage revision for the culture-negative infected total hip arthroplasty.	Culture-negative periprosthetic joint infection does not preclude infection control.	Culture-negative Prosthetic Joint Infection.	Prior Use of Antimicrobial Therapy is a Risk Factor for Culture-negative Prosthetic Joint Infection.	Comparison of infection control rates and clinical outcomes in culture-positive and culture-negative infected total-knee arthroplasty.	The outcome of infected total knee arthroplasty: culture-positive versus culture-negative.

Year	2017	2013	2017	2012	2007	2010	2015	2015

Country	Netherlands	United States	UK	United States	United States	United States	Korea	Korea

LoE	III	III	III	III	III	III	III	III

Study design	Retrospective	Retrospective	Prospective	Retrospective	Retrospective	Retrospective	Retrospective	Retrospective

Study Type	Case-Control study	Case-Control study	Case-Control study	Case-Control study	Cohort study	Case-Control study	Case-Control study	Case-Control study

Treatment interval	2003-2014	2000-2009	2007-2012	2000-2007	1990-1999	1985-2000	2001-2008	1991-2008

Total number of cases	129	175	-	295	897	1413	242	191

Prevalence of CN cases %	14.2	23	-	16.3	7	10.5	42.1	26.7

Hip %	-	50	100	43.8	45	50	-	-

Knee %	100	50	-	56.2	55	50	100	100

FU in months, median	55.6	52	60	47	36-60	56	127.2	127.2

Risk factors		-	(1) prior use of antibiotics (2) referral from elsewhere (3) age		(1) prior use of antibiotics (within 3 months)	(1) prior use of antibiotics (in 64%) (2) prolonged wound drainage after index arthroplasty (residual confounder)	-	-

Debridement n	-	11	-	12	12	18	56	28

1-stage n				3	5	8	-	-

2-stage n	18	23	50	33	34	56	46	23

Permanent resection n		-	-	-	8	34	-	-

Other therapy		6	-	-	1	19	-	-

Antibiotic treatment after diagnosis %	Vancomycin 33 Vancomycin + Ceftriaxone 33 Others 34	Vancomycin 70; Others 30	-	Vancomycin 81; Cephalosporins 10; Others 9	Cephalosporins 82; Vancomycin 12; Others 6	Cefazolin 69; Vancomycin 13; Others/None 18	Vancomycin 85; Others 15	Vancomycin 86; Others 14

Successful treatment in %	88,9	85	94	-	-	-	95	95

Overall infection free survival rate % 1.) 3-year 2.) 5-year	-	-	1.) - 2.) 94	73	-	1.) - 2.) 67	-	-

I&D infection free survival rate % 1.) 3-year 2.) 5-year	-	-	-	50	1.) - 2.) 71	78	57	61

2-Stage infection free survival rate % 1.) 3-year 2.) 5-year	1.) 75 2.) 95	-	-	58	1.) - 2.) 94	1.) 87 2.) 79	83	83

1-Stage infection free survival rate % 1.) 3-year 2.) 5-year	-	-	-	100	-	-	-	-

Resection arthroplasty infection free survival rate % 1.) 3-year 2.) 5-year	-	-	-	-	1.) 51	1.) 49 2.) 43	-	-

Outcome	With combined or broad-spectrum antibiotics, two-stage revision showed comparable outcome in satisfaction rates, reinfections rates and cumulative survival rates at 5-year Follow-up with CP PJI patients.	The success rate of infection control was higher in the CN group, which suggests that CN may not necessarily be a negative prognostic factor for PJI.	-	The overall infection control rate was similar between CP and CN PJI cases (both 73%).	The outcome of CN PJI is similar to the outcome of PJI due to known pathogens.	The demographics and outcome of CP and CN PJI patients were similar (free of treatment failure at 2 years 79% and 75%).	The infection control rates and clinical outcomes were not different between CP and CN groups (overall infection control rates 90% and 95%).	Overall rates of infection control, successful treatment, and functional outcomes were not different between the CP and CN groups (overall infection control rates 90% and 95%).

**Table 3:** Different diagnostic criteria for periprosthetic joint infections [3–13].

Parameter	MSIS criteria	AAOS	Philadelphia Consensus	Parvizi et al.	Aggarwal et al. [3]	Zimmerli et al.	Trampuz et al.	Tohtz et al. [11]	Atkins et al.	Portillo et al.	Shanmugasundaram et al. [9]	Müller et al.	Spangehl et al. [10]	Tohtz et al.	Aggarwal et al.	Shanmugasundaram et al.	Spangehl et al.	Charité (modified from Zimmerli)
**Clinical**																		
Sinus tract or abscess	x	x	x	x	x	x	x	x	x	x	x		x	x	x	x	x	x
Pain or poor functional status					x						x	x	x			x	x	
Erythema or swelling					x						x	x			x	x		
Pus												x	x	x	x	x	x	
Early loosening												x						
**Labor**																		
CRP	x (>10mg/L)		x	x (>10mg/L)	x (>10mg/L)			x (>10mg/L)				x (>0,5mg/dl)	x (>10mg/L)	x (>10mg/L)	x (>10mg/L)		x (>10mg/L)	x
ESR	x (>30mm/h)		x	x (>30mm/h)	x (>30mm/h)			x (>30mm/h)				x	x (>30mm/h)	x (>30mm/h)	x (>30mm/h)		x (>30mm/h)	x
Leucocytes								x (>10.000/ul)				x (>12.000/ul)		x (>10.000/ul)				
**Histology**																		
Acute infection	x	x	x	x		x	x	x	x	x	x	x	x					x
Acute infection (Type II or III according to Krenn/Morawietz)								x			x			x (>2)				
Granulocytes/HPF	x (>5)			x (>5)		x (1-10)	x (≥10 )	x (≥2 )	x (>5)		x (≥10 )	x (≥2 )	x (>5)	x (>2)		x (≥10 )	x (>5)	
**Microbiology**																		
≥2 culture-positive samples	x	x	x	x		x	x	x	x (≥3)	x	x	x		x		x	x	x
1 culture-positive sample	x		x	x			x						x				x	x
1 culture-positive sample (high virulent germ)		x																x
**Sonication**																		
≥ 50 colonies/ml																		
**Synovial flood**																		
Leucocytes	x (>1.100 cells/ul)		x	x (>1.100 cells/ul)	x	x (>1.700 cells/ul)	x (>1.700 cells/ul)			x (>1.700 cells/ul)					x			x
Granulocytes	x (>64%)		x	x (>64%)	x	x (>65%))	x (>65%))			x (>65%))					x			x
Pus	x	x		x	x	x		x	x	x	x	x	x			x		x
Culture positive		x		x	x		x					x	x		x		x	
Main criteria																		
Secondary criteria																		

**Table 4:** Diagnostic parameters for CN PJI [23].

**Test**	**Criteria**	**Sensitivity**	**Specificity**
**Clinical features**	Sinus tract (fistula) **or **purulence around prosthesis^a^	20-30%	100%

**Leukocyte count in synovial fluid** ^b^	>2000/ul leucocytes **or **>70% granulocytes (PMN)	≈90%	≈95%

**Periprosthetic tissue histology** ^c^	Inflammation (≥23 granulocytes per 10 high-power fields)	73%	95%

**Microbiology**	Microbial growth in: (i) synovial fluid or (ii) ≥2 tissue samples^d^ or (iii) sonication fluid (>50 CFU/ml)^e^	45-75% 60-80% 80-90%	95% 92% 95%

^a^Metal-on-metal bearing components can simulate pus (≪pseudopus≫), leukocyte count is usually normal (visible is metal debris)

^b^Leukocyte count can be high without infection in the first 6 weeks after surgery, in rheumatic joint disease (including crystalopathy), periprosthetic fracture or luxation.

Leukocyte count should be determined within 24 h after aspiration by microscopy or automated counter; clotted specimens are treated with 10 *μ*l hyaluronidase

^c^Classification after Krenn and Morawietz: PJI corresponds to type 2 or type 3

^d^For highly virulent organisms (e.g. *S. aureus*, streptococci, *E. coli*) or patients under antibiotics, already one positive sample confirms infection

^e^Under antibiotics, for *S. aureus *and anaerobes, <50 CFU/ml can be significant

**Table 5:** Antimicrobial treatment in CN PJI [23].

**Microorganism**	**Antibiotic** ^**a**^	**Dose** ^**b**^	**Route **
(red: difficult-to-treat)	(check pathogen susceptibility before)	(italic font: *renal adjustment needed*)	
**Culture-negative**	Ampicillin/sulbactam^c^	*3 × 3 g*	i.v.
	for 2 weeks, followed by:		
	Rifampin^d^ + Levofloxacin	2 × 450 mg	p.o.
		*2 × 500 mg*	p.o.

^a^ **Total duration** of therapy: **12 weeks**, usually 2 weeks intravenously, followed by oral route.

^b^Laboratory testing 2x weekly: leukocytes, CRP, creatinine/eGFR, liver enzymes (AST/SGOT and ALT/SGPT). Dose-adjustment according to *renal function* and body weight (<40/> 100kg).

^c^ **Penicillin allergy** of NON-type 1 (e.g., skin rash): cefazolin (3 × 2 g i.v.). In case of anaphylaxis (= type 1 allergy such as Quincke's edema, bronchospasm, and anaphylactic shock) or cephalosporin allergy, vancomycin (2 × 1 g i.v.) or daptomycin (1 × 8 mg/kg i.v.).

Ampicillin/sulbactam is equivalent to amoxicillin/clavulanic acid (3 × 2.2 g i.v.).

^d^ **Rifampin** is administered only after the new prosthesis is implanted. Add it already to intravenous treatment as soon as wounds are dry and drains removed; in patients aged >75 years, rifampin is reduced to 2 × 300 mg p.o.

## Appendix

See Table 1 and Figure 4.
